# Neutrophil in sepsis: functional aberration and regulated cell death

**DOI:** 10.3389/fimmu.2026.1863084

**Published:** 2026-06-05

**Authors:** Li Liu, Yanli Yang, Haixia Yang, Meng Yang

**Affiliations:** 1 Department of Neonatology, Jinan Maternity and Child Care Hospital Affiliated to Shandong First Medical University, Jinan, China; 2 Operating Room, Public Health Clinical Center Affiliated to Shandong University, Jinan, China

**Keywords:** functional dysregulation, immunomodulation, neutrophils, regulated cell death, sepsis

## Abstract

Sepsis is defined as life-threatening organ dysfunction caused by a dysregulated host response to infection and is a leading cause of death in critically ill patients. Studies have shown that immune dysfunction plays a central role in the pathogenesis of sepsis. As key effector cells of innate immunity, neutrophils play an important role in the development of sepsis-associated immune dysfunction. In the septic state, neutrophils mainly exhibit functional abnormalities and aberrant activation of cell death pathways. Among these, the inhibition of neutrophil apoptosis is considered a major contributor to excessive inflammatory responses. Meanwhile, with continuous advances in research, other forms of neutrophil cell death such as necroptosis, pyroptosis, and NETosis have also been confirmed to be closely associated with the onset and progression of sepsis. Over the years, various strategies have been devised and effectively implemented to ameliorate aberrant immune responses during the progression of sepsis, including modulation of neutrophil function and death. This review will outline the functional alterations and cell death patterns of neutrophils in the septic state, with a focus on recent research advances in targeting neutrophils to regulate the host immune response following septic challenge, aiming to provide new insights for the treatment of sepsis.

## Introduction

Sepsis is a life-threatening clinical complication frequently encountered in patients with trauma, burns, infection, or shock. It is characterized by rapid progression and high mortality, and has long been regarded as a major challenge in critical care medicine (1, 2). In the context of public health emergencies such as the COVID-19 pandemic, sepsis and its consequent multiple organ dysfunction syndrome have further exacerbated the burden on global healthcare systems (1). A multicenter study in Brazil revealed that the incidence of sepsis in intensive care units reached 33%, with an associated in-hospital mortality rate of 55.7% (3). A 2017 global survey reported nearly 48.9 million annual sepsis cases, resulting in 11 million deaths (19.7% of global mortality). This figure substantially exceeds prior estimates derived mainly from hospitalized patients in high-income countries (4). Current evidence suggests that the actual disease burden of sepsis may be more severe than previously recognized. Therefore, elucidating the molecular mechanisms and cellular dysfunctions underlying sepsis, along with exploring key therapeutic targets, is of great scientific and clinical importance for optimizing treatment strategies and improving patient outcomes.

Neutrophils constitute the most abundant leukocyte population in the peripheral circulation of healthy adults, accounting for 50%–70% of all circulating white blood cells, and play a critical role in recognizing and eliminating bacterial and fungal pathogens (5). Current evidence indicates that neutrophils exert multidimensional regulatory functions in the pathological process of sepsis. Traditional views hold that they primarily control infection and ameliorate sepsis by eliminating pathogens through phagocytosis (6). However, recent studies have revealed that neutrophils are deeply involved in the occurrence, development, and clinical outcome of sepsis through mechanisms including exacerbating inflammatory responses, inducing immune suppression, and participating in the coagulation cascade (7–9). Specifically, during sepsis, neutrophils exhibit not only aberrant functional activation but also altered cell death patterns. For instance, persistent septic stimulation induces extensive neutrophil migration to organs (10). Concurrently, significant upregulation of the anti-apoptotic protein myeloid cell leukemia 1 (MCL-1) in neutrophils inhibits apoptosis and prolongs their half-life (11). Although the increased chemotaxis and extended lifespan enable neutrophils to perform more complex activities in tissues, their massive accumulation and persistence can induce fatal inflammatory responses and exacerbate organ damage by releasing large quantities of damage-associated molecular patterns (DAMPs) and reactive oxygen species (ROS), representing a major factor contributing to the high mortality rate in sepsis (10). Furthermore, under lethal inflammatory conditions, the activation of inflammatory cell death pathways (e.g., pyroptosis, necroptosis, and NETosis) in neutrophils can further aggravate cell death and amplify inflammatory factor release, leading to a cascade that ultimately intensifies systemic inflammation and accelerates sepsis progression. Notably, with advanced high-throughput analytical techniques such as single-cell RNA sequencing (scRNA-seq) and cytometry by time-of-flight (CyTOF), recent studies have identified multiple novel neutrophil subpopulations associated with sepsis (12–14). These previously overlooked neutrophil subsets possess unique functions and play critical roles in the pathology of sepsis. For example, a recent study discovered a subset of S100 calcium binding protein A8/A9^high^ (S100A8/A9^hi^) neutrophils in lung tissues during sepsis, which can induce PANoptosis of endothelial cells by releasing large amounts of S100A8/A9, thereby exacerbating sepsis-induced lung injury (14).

Given the critical pathophysiological significance of neutrophil dysfunction in the development and progression of sepsis, this article systematically summarizes the roles of neutrophil functional dysregulation and newly identified functional subsets in the pathogenesis of sepsis, aiming to provide an in-depth analysis of the pathological mechanisms and identify potential targets for immunomodulatory therapy. Furthermore, recent research has developed new strategies that target neutrophils by precisely controlling their programmed cell death. Such interventions have shown significant potential to reduce mortality in sepsis and septic shock cases. Based on this, this review will also systematically elaborate on the latest research progress of neutrophil-related programmed cell death during sepsis, focusing on analyzing the molecular regulatory mechanisms of different death modes (e.g., apoptosis, necroptosis, pyroptosis, and NETosis), with the goal of providing a new theoretical framework for sepsis treatment.

## The functional dysregulation of neutrophil in sepsis

**Figure 1:**
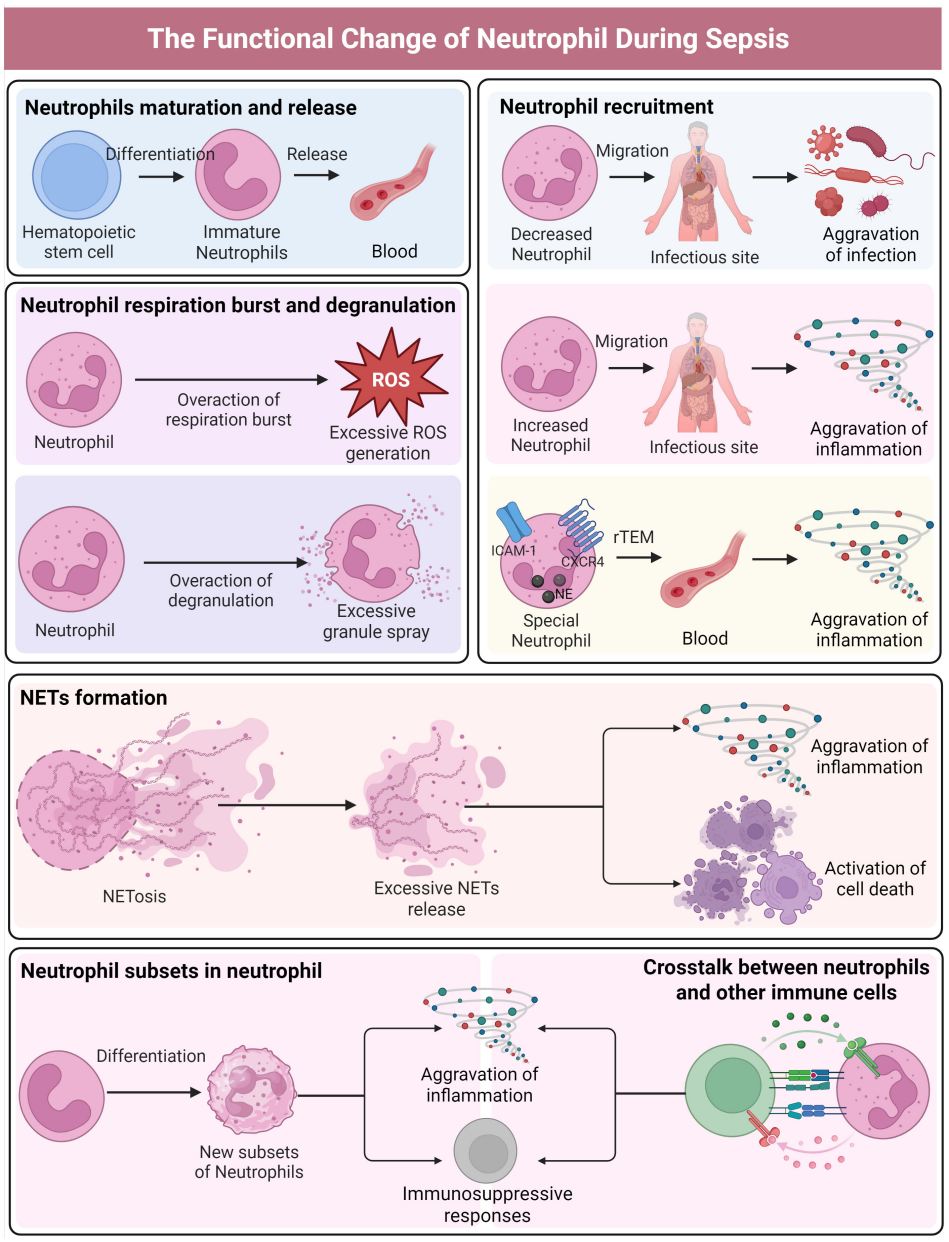
The functional change of neutrophil during sepsis. Summarizing the functional changes of neutrophils during sepsis, including neutrophil maturation and release, neutrophil recruitment, neutrophil respiratory burst and degranulation, NETs formation, neutrophil subsets in sepsis, and crosstalk between neutrophils and other immune cells.

Neutrophils are widely recognized as key drivers of sepsis pathogenesis, a life-threatening condition in which their functional state undergoes profound and dynamic alterations. In response to septic stimuli, these cells exhibit a broad spectrum of functional changes that critically influence disease progression and host defense (5). Research in this field has increasingly focused on characterizing the functional landscape of neutrophils under septic conditions, with six key aspects emerging as particularly important for understanding their dual role in both pathogen clearance and immunopathology (**Figure 1**).

## Neutrophil maturation and release

As essential phagocytes in the host defense system, neutrophils rapidly migrate from the circulation to infection or inflammation sites upon pathogenic challenge. These cells are characterized by their large numbers, rapid turnover, and short lifespan (5). Under homeostatic conditions, mature neutrophils are generated from hematopoietic stem cells (HSCs) through a multi-stage differentiation process involving various progenitors and precursors in the bone marrow (BM), before being released into the peripheral circulation (15). During sepsis, the body significantly increases neutrophil production via “emergency granulopoiesis,” leading to the release of both mature and immature neutrophils into the bloodstream to meet physiological demands (16). These immature neutrophils exhibit notable functional impairments, including reduced recognition and phagocytic capacity, diminished deformability, and a tendency to lodge in capillaries, contributing to microthrombosis, vascular obstruction, tissue hypoxia, and organ damage (17, 18). Mature neutrophils display a distinct surface marker profile, characterized as CD11b^+^Ly6G^+^CXCR2^+^CD101^+^. In contrast, immature neutrophils lack expression of chemokine C-X-C motif receptor 2 (CXCR2) and CD101 (19, 20). Consequently, some researchers have proposed that the proportion of immature neutrophils among total peripheral neutrophils could serve as a potential biomarker for sepsis diagnosis, but its clinical utility requires further validation through large-scale studies (20). Specifically, the migration of neutrophils in the bone marrow depends on the dynamic balance between retention signals and release signals. Among these, CXCR4 and CXCR2 are the two most critical markers controlling this process (21). Under physiological conditions, only a small number of mature neutrophils enter the peripheral circulation. By upregulating CXCR4 and downregulating CXCR2, circulating senescent neutrophils enable their return to the bone marrow for clearance by macrophages (21). In sepsis, the CXCR4/C-X-C motif ligand 12 (CXCL12) signaling axis is suppressed, while the CXCR2/CXCL1 axis is markedly activated, thereby accelerating the release of neutrophils from the bone marrow into the circulation (22). Notably, during this process, pro-inflammatory cytokines and bacterial products can upregulate granulocyte colony-stimulating factor (G-CSF), which in turn induces neutrophil maturation and release. Mechanistically, G-CSF promotes the proliferation and differentiation of CD34^+^ granulocyte progenitors and facilitates the release of mature neutrophils by inhibiting the CXCR4/CXCL12 axis (22). Notably, studies have found that the loss of CD11c in neutrophil precursors during sepsis exacerbates the release of immature neutrophils into the circulation, accompanied by massive proliferation and increased apoptosis of precursor neutrophils (23). In contrast, septic mice with CD11c knock-in exhibit accelerated neutrophil maturation and enhanced effector function, suggesting that CD11c is a key molecule regulating neutrophil maturation. Targeted regulation of CD11c activation in neutrophils may represent a new direction for sepsis treatment (23).

## Neutrophil recruitment: from targeted chemotaxis to aberrant migration

**Figure 2:**
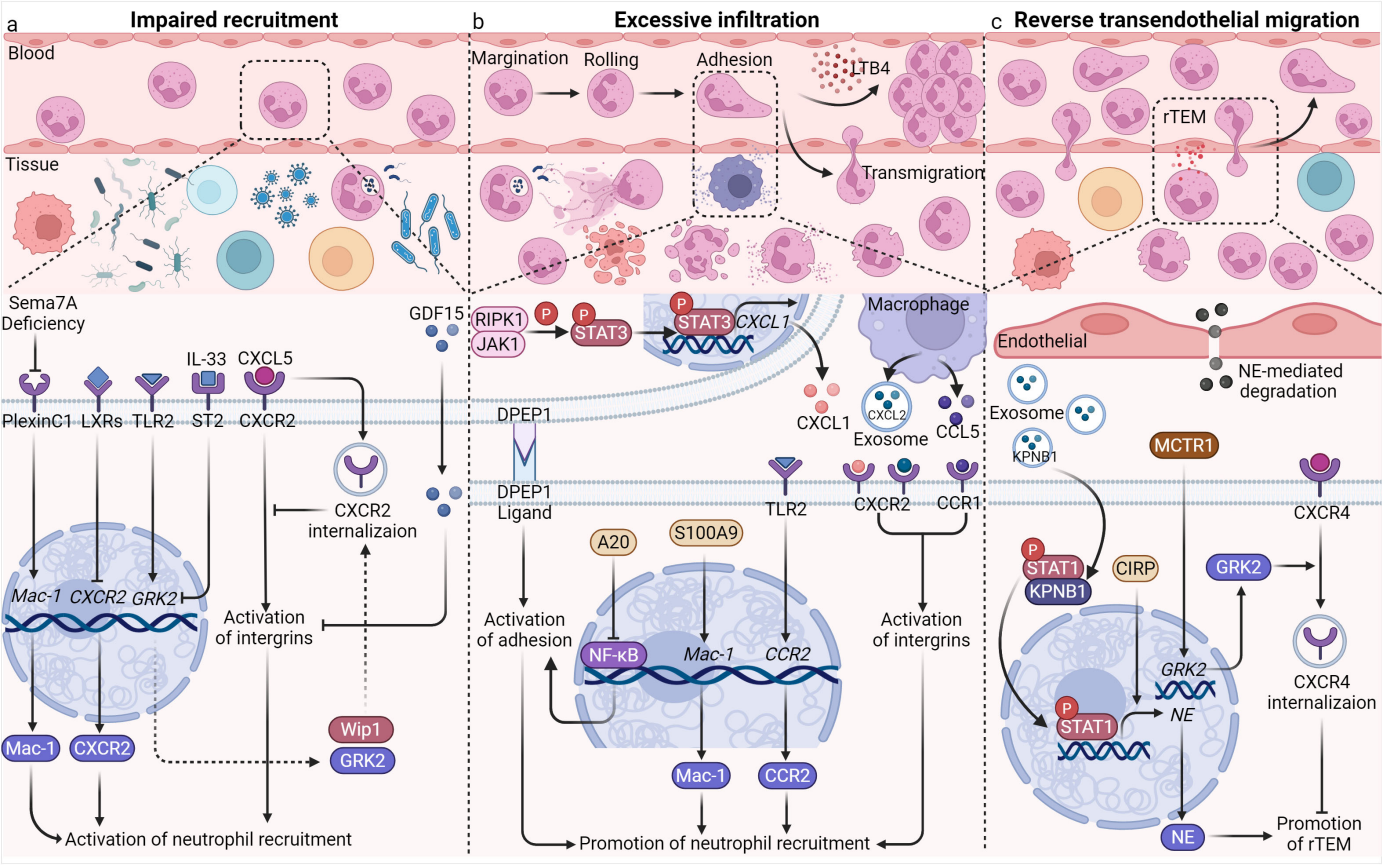
Dysregulation of neutrophil recruitment in sepsis. **(a)** Sema7A deficiency inhibits PlexinC1 receptor activation to suppress Mac-1 expression and impair neutrophil recruitment. GDF15 inhibits CXCL5-CXCR2 axis activation to impair neutrophil recruitment. LXRs downregulates CXCR2 to inhibit neutrophil recruitment. TLR2 upregulates GRK2 expression to promotes CXCR2 internalization and impair neutrophil recruitment. Wip1 promotes CXCR2 internalization to inhibit neutrophil migration. IL-33-ST2 signaling axis downregulates GRK2 expression to promotes CXCR2 internalization and impair neutrophil recruitment. **(b)** LTB4 induces neutrophil aggregation and vascular occlusion, triggering pulmonary capillaritis. DPEP1 functions as a physical adhesion receptor to promote neutrophil recruitment. RIPK1 interacts with JAK1 to promote STAT3 phosphorylation and nuclear translocation, leading to CXCL1 upregulation and excessive neutrophil recruitment. Macrophage secrete CCL5 chemokine to activate CCL5-CCR1 signaling axis and promote neutrophil recruitment. Exosomes-derived macrophage activate CXCL2-CXCR2 axis to promote neutrophil recruitment. A20 protein deficiency promotes NF-kB to promote neutrophil adhesion and migration. S100A9 upregulates Mac-1 expression to promote neutrophil recruitment. TLR2 activation upregulates CCR2 expression to promote neutrophil recruitment. **(c)** Exosomes-enriched KPNB1 promotes STAT1 phosphorylation and nuclear translocation, leading to upregulation of NE and promotion of neutrophil rTEM. CIRP promotes NE expression to increase neutrophil rTEM. MCTR1 upregulates GRK2 to promote CXCR4 endocytosis and inhibit neutrophil rTEM.

Neutrophil recruitment refers to the directed migration of circulating neutrophils to sites of infection or inflammation (5, 10). This process involves multiple steps, including activation of vascular endothelial cells, as well as rolling, adhesion, migration, and chemotactic movement of neutrophils toward inflammatory foci (10). Specifically, inflammatory mediators not only induce the expression of E-selectin, P-selectin, and chemokines (e.g., CXCL8) on endothelial cells but also upregulate glycoproteins (e.g., P-selectin glycoprotein ligand 1 [PSGL-1]) and chemokine receptors (e.g., CXCR1/CXCR2) on circulating neutrophils. During rolling, neutrophils use surface glycoproteins to interact with selectins under shear stress. Simultaneously, chemokines secreted by endothelial cells bind to their receptors on neutrophils, leading to the activation of integrins (lymphocyte function-associated antigen-1 (LFA-1), macrophage-1 antigen (Mac-1)) (10). The activated integrins then strongly bind to intercellular adhesion molecule-1 (ICAM-1)/ICAM-2 on the endothelial surface, causing neutrophils to cease rolling and adhere firmly. Finally, neutrophils traverse the endothelial gaps along a chemotactic gradient (e.g., interleukin [IL-8], C5a, leukotriene B4 [LTB4]) and migrate into infected or injured tissues (10). Substantial evidence indicates that dysregulated neutrophil recruitment plays a critical role in the pathogenesis and progression of sepsis. The main aberrant phenotypes include impaired recruitment, excessive infiltration, and reverse transendothelial migration (**Figure 2**).

### Impaired recruitment and defective host defense

A study has found that the serum level of growth differentiation factor 15 (GDF15) is significantly elevated in patients with septic shock, and its expression level is positively correlated with patient mortality. Animal experiments have confirmed that targeted intervention in GDF15 expression can effectively attenuate systemic inflammatory responses and significantly improve the survival rate of sepsis model mice (24). Mechanistically, GDF15 exacerbates sepsis-related infection and injury by inhibiting the activation of the CXCL5-CXCR2 signaling axis, reducing neutrophil recruitment to infection sites, and thereby impairing pathogen clearance efficiency (24). Ferreira et al. discovered that galectin-3 can exacerbate lung infection by inhibiting neutrophil migration, thereby inducing lung injury in sepsis. Notably, a cohort study of sepsis patients revealed that serum galectin-3 levels are significantly higher in shocked patients compared to non-shocked patients (25). The deficiency of Semaphorin 7A (Sema7A) has been found to inhibit PlexinC1 (PLXNC1) receptor activation, resulting in the downregulation of Mac-1 expression, impaired migration of neutrophils to lung tissue, and ultimately aggravated lung infection (26). Activation of liver X receptors (LXRs) has been confirmed to significantly inhibit neutrophil migration to infection sites by downregulating CXCR2 and inhibiting the activation of the CXCL8-CXCR2 signaling axis, thereby exacerbating sepsis progression (27). NOD-like receptor family pyrin domain-containing protein 6 (NLRP6) has been found to be closely associated with neutrophil chemotaxis during sepsis. NLRP6 knockout mice exhibit reduced pulmonary neutrophils, accumulation of mature bone marrow neutrophils, and increased infection burden in pneumonia-derived sepsis, but the specific mechanism remains to be further elucidated (28). Additionally, it has been reported that CXCL1 deficiency significantly inhibits neutrophil migration to the lungs, leading to impaired pathogen clearance and ultimately accelerating the pathological progression of pneumonia-derived sepsis (22). Another study found that in diabetic mouse infection models, glucagon can weaken the host’s anti-infective immune response by inhibiting CXCL1-mediated neutrophil chemotaxis, thereby increasing sepsis susceptibility (29). Notably, CXCR2 internalization refers to the process by which CXCR2 receptors on the cell membrane enter the cytoplasm through endocytosis, resulting in a reduction in the number of membrane-bound receptors, and is a key link in chemokine signal regulation (10). Shen et al. found that pharmacological inhibition or genetic knockout of Wild-type p53-induced phosphatase 1 (Wip1) protein in neutrophils blocked CXCR2 internalization. This intervention maintained high surface CXCR2 levels, enhanced neutrophil sensitivity to chemokines, promoted infiltration into infected tissues, and improved outcomes in septic mice (30). It was reported that, during sepsis, Toll-like receptor 2 (TLR2) activation can accelerate CXCR2 endocytosis by upregulating G protein-coupled receptor kinase 2 (GRK2) expression in neutrophils, inhibiting neutrophil migration and impairing infection clearance (31). In contrast, IL-33/ST2 signaling suppresses GRK2 expression, reverses TLR4-induced CXCR2 internalization, and enhances neutrophil migration and bacterial clearance (32).

### Excessive infiltration and organ dysfunction

Excessive neutrophil chemotaxis during sepsis is a critical pathological feature, and its driving factors can be summarized into the following three main aspects. First, vascular dysfunction. A study has found that dipeptidase-1 (DPEP1) functions as a physical adhesion receptor on hepatic and pulmonary vascular endothelial cells, mediating excessive neutrophil migration to the liver and lung tissues during sepsis (33). Targeted inhibition of DPEP1 expression blocks abnormal neutrophil infiltration into lungs and liver, significantly ameliorating sepsis-induced organ damage (33). Through interaction with Janus kinase 1 (JAK1), receptor-interacting protein kinase 1 (RIPK1) in type II alveolar epithelial cells can promote signal transducer and activator of transcription-3 (STAT3) phosphorylation, nuclear translocation, and binding to the CXCL1 promoter, leading to CXCL1 upregulation and excessive neutrophil recruitment (34). In a *Staphylococcus aureus*-induced sepsis mouse model, the structure of the vascular glycocalyx (VGC) in the liver is significantly disrupted. This pathological change promotes the abnormal migration of neutrophils to the liver, thereby exacerbating liver injury. Notably, precise regulation of the molecular structure of heparan sulfate (HS) in the vascular glycocalyx can effectively reverse abnormal neutrophil infiltration, significantly alleviating vascular inflammatory responses and secondary liver injury (35). Second, dysregulated activation of macrophages. Fan et al. discovered that during sepsis, chemokine (C-C motif) ligand 5 (CCL5)^+^ macrophages in the kidneys can continuously secrete CCL5 chemokine, activating the CCL5-chemokine-receptor 1 (CCR1) signaling axis, accelerating the directed migration of neutrophils to kidney tissues and ultimately aggravating sepsis-related kidney injury (36). Another study reported that macrophages drive the abnormal migration of neutrophils to the liver by releasing exosomes enriched in CXCL2. Specific blocking of this exosome secretion pathway can effectively reduce hepatic neutrophil infiltration, thereby significantly improving sepsis-related liver injury (37). Third, functional dysregulation of neutrophils. A20 protein has been found to inhibit neutrophil adhesion to endothelium in sepsis through negative regulation of nuclear factor kappaB (NF-κB) signaling (38). This reduction in adhesion limits pathological neutrophil infiltration into organs and significantly enhances survival in neonatal mouse sepsis models (38). S100A9 can significantly enhance neutrophils recruitment by upregulating the expression of Mac-1 in neutrophils, thereby exacerbating sepsis-induced lung injury, but the specific mechanism remains to be further elucidated (39). Deficiency of hematopoietic cell-specific Lyn substrate (HS1) protein exerts a dual protective effect against sepsis by precisely regulating the dynamic balance of neutrophil recruitment: it retains the basal level of neutrophil recruitment required for pathogen clearance while effectively preventing tissue damage caused by excessive infiltration (40). Lee et al. found that in fungal sepsis, LTB4 promotes the aggregation of intravascular neutrophils and vascular occlusion, triggering pulmonary capillaritis, which leads to pulmonary hemorrhage and hypoxemia (41). Interestingly, contrasting with the above findings, some researchers have found that TLR2 activation during sepsis upregulates CCR2 expression on neutrophils, driving their abnormal migration to multiple organs and ultimately contributing to sepsis-associated multiple organ dysfunction syndrome (MODS) (42). These results indicate that TLR2 regulates neutrophil migration in a bidirectional manner: on the one hand, TLR2 activation suppresses neutrophil migration by upregulating GRK2 expression; on the other hand, TLR2 also leads to excessive neutrophil infiltration into organs via upregulation of CCR2 expression (31, 42). This bidirectional property suggests that in the treatment of sepsis, the activation status of the TLR2 pathway could be used to assess patient conditions and guide targeted interventions. Furthermore, further elucidation of the molecular mechanisms underlying TLR2-mediated bidirectional neutrophil migration may provide a theoretical basis for developing new therapeutic strategies.

### Reverse transendothelial migration

It has been established that a specific neutrophil subpopulation characterized by high ICAM-1/CXCR4 and low CXCR1 expression can re-enter the bloodstream from initial inflammatory sites via an “extravascular-to-intravascular” route, a process termed neutrophil reverse transendothelial migration (rTEM) (43, 44). Neutrophil elastase (NE), a key effector molecule released by neutrophil degranulation, can drive the occurrence of neutrophil rTEM by specifically degrading junctional adhesion molecule-C (JAM-C) (45). Recent studies have revealed that the proportion of rTEM neutrophils in peripheral blood is significantly higher in sepsis patients with acute respiratory distress syndrome (ARDS) than in those with sepsis alone, and correlates positively with lung injury (46). Mechanistically, endothelial cells secrete exosomes containing karyopherin subunit beta-1 (KPNB1), which activate the STAT1 signaling pathway in neutrophils and significantly upregulate NE expression. NE subsequently degrades endothelial JAM-C, promoting rTEM and facilitating neutrophil translocation to the lungs. This cascade significantly exacerbates sepsis-induced acute lung injury (46). Jin et al. also found that cold-inducible RNA-binding protein (CIRP) promotes NE expression during sepsis, leading to disruption of vascular JAM-C and increased neutrophil rTEM (47). In addition, macrophage-derived maresin conjugates in tissue regeneration 1 (MCTR1) has been confirmed to inhibit neutrophil rTEM by upregulating GRK2, promoting CXCR4 endocytosis, thereby alleviating lung injury during sepsis (48).

In summary, neutrophil responses in sepsis are not static but follow a distinct temporal trajectory. In the early phase, enhanced chemotaxis, phagocytosis, and NET formation contribute to pathogen clearance and host defense. As sepsis progresses, however, neutrophils undergo a phenotypic shift toward pathological activation, characterized by excessive infiltration, dysregulated respiratory burst, and aberrant activation of cell death pathways. This transition from protective to detrimental function underscores that the timing of therapeutic intervention is critical: early modulation to preserve neutrophil function may improve infection control, whereas late suppression of overactivation may limit tissue injury. Therefore, how to precisely regulate the dynamic balance of neutrophil migration—maintaining effective pathogen clearance while avoiding excessive inflammatory injury—remains a key scientific challenge in the current field of sepsis treatment.

## Neutrophil respiratory burst and degranulation

Neutrophils eliminate pathogens through both oxygen-dependent and oxygen-independent pathways (49). In the oxygen-dependent pathway, the nicotinamide adenine dinucleotide phosphate (NADPH) oxidase complex assembles on the phagosomal membrane, catalyzing the production of large amounts of superoxide anions and ROS (49). ROS exert bactericidal effects by oxidatively damaging bacterial cell membranes, nucleic acids, and proteins. Simultaneously, ROS can upregulate the expression of cytokines and adhesion molecules, amplifying inflammatory responses and potentially contributing to cytokine storms. During this process, neutrophil oxygen consumption surges to 2–20 times baseline levels, a phenomenon termed the “respiratory burst” (50). Oxygen-independent killing by neutrophils primarily relies on molecular mechanisms mediated by their cytoplasmic granules. Based on their contents, these granules are classified into four subtypes: azurophilic granules, specific granules, tertiary granules, and secretory granules. These granules contain various bactericidal active proteins, including myeloperoxidase (MPO), neutrophil serine proteases (NSPs), cathepsin G, and NE (49, 50). Through complex synergistic effects, these granule contents not only form an important innate immune barrier against pathogenic microbial invasion but also play critical biological roles in regulating the progression of inflammatory responses (49, 50).

In sepsis, infiltrating neutrophils generate large amounts of ROS via the respiratory burst and release bactericidal substances through degranulation. However, high concentrations of oxygen free radicals produced during the respiratory burst can disrupt cellular homeostasis through multiple pathways, including: induction of mitochondrial dysfunction (51); promotion of neutrophil extracellular traps (NETs) formation and release (52, 53); and activation of inflammatory signaling pathways (54, 55). These pathological alterations collectively form an important molecular basis for sepsis progression. Furthermore, neutrophil degranulation plays a critical role in the pathophysiology of sepsis. Through specific cleavage of sialic acid-binding immunoglobulin-type lectin-G (Siglec-G) on peritoneal B-1a cells, NE disrupts the Siglec-G/CXCL12/CXCR4 complex. This relieves the negative regulation on CXCL12-CXCR4 signaling, thus promoting aberrant B-1a cell migration and aggravating sepsis progression (56). A recent study showed that patients with sepsis-associated lymphopenia have significantly higher peripheral blood levels of MPO and NE compared to non-lymphopenic patients, with both enzyme levels exhibiting a significant positive correlation with the extent of CD4^+^ T cell pyroptosis (57). Okeke et al. identified the inhibition of NE release as an effective strategy to reduce NETs formation and attenuate septic shock (58). Additionally, it has been reported that the abnormal elevation of NE in sepsis can accelerate the destruction of the pulmonary endothelial glycocalyx, thereby exacerbating pulmonary vascular leakage and inflammatory responses, and ultimately worsening sepsis-induced lung injury (59). Notably, NE exhibits a contradictory dual role in sepsis-induced kidney injury. Zhu et al. found that in a rat model of CLP-induced sepsis without penicillin intervention, targeted inhibition of the NE-oxidative stress axis represents a novel renal protective strategy, with the core mechanism involving the maintenance of the dynamic balance of the renal antioxidant enzyme system (60). In contrast, another study demonstrated that in a rat sepsis model treated with penicillin after CLP surgery, NE serves as a key mediator of dexamethasone (DEX)-induced renal protection, alleviating kidney injury by suppressing renal inflammatory responses (61). However, the specific targets and downstream signaling pathways of NE in this process require further investigation.

## Neutrophil extracellular traps formation

Neutrophils capture and eliminate unphagocytosed pathogens by releasing NETs (62). This process, termed NETosis, represents a distinct form of programmed cell death that differs fundamentally from other cell death pathway (63). Structurally, NETs are composed of a backbone of DNA fibers decorated with various effector molecules, including histones, granule−derived enzymes, and multiple antimicrobial peptides (62). A wide range of stimuli can trigger NET formation, including pathogen−associated molecular patterns (PAMPs), proinflammatory cytokines (e.g., IL−6 and IL−8), antigen−antibody complexes, and activated platelets (64).

During the early stages of sepsis, NETs serve a protective function by forming physical barriers that trap and immobilize pathogens, thereby limiting their dissemination and promoting local containment of infection (62). However, the protective role of NETs is context−dependent. Under dysregulated or excessive conditions, NETs can also act as DAMPs, activating cell surface or intracellular pattern recognition receptors such as TLR9. This activation triggers downstream inflammatory signaling pathways, induces cell death, and exacerbates tissue injury (64). Accumulating evidence indicates that NETs released during sepsis are critical drivers of disease progression and multi−organ failure, largely through their capacity to activate diverse programmed cell death pathways in both immune cells and parenchymal cells (64). The detailed molecular mechanisms underlying these NET−mediated effects will be explored in depth in the following sections.

## Neutrophil subsets in sepsis

Recent technological breakthroughs, particularly in single-cell sequencing and high−dimensional flow cytometry, have revolutionized our understanding of neutrophil biology. These advanced analytical tools have unveiled the remarkable heterogeneity of neutrophils during the pathological progression of sepsis, revealing that these cells are far from a homogeneous population. Instead, distinct neutrophil subsets exist, each contributing to disease pathogenesis through specific molecular and functional mechanisms (12–14). Given this complexity, a thorough investigation into the developmental origins, phenotypic diversity, and functional characteristics of these subsets is essential. Such efforts will facilitate the establishment of novel disease monitoring indicators that reflect the dynamic shifts in neutrophil subset composition over the course of sepsis. Moreover, a deeper understanding of subset−specific roles may pave the way for the development of precision therapeutic strategies that selectively target pathogenic neutrophil subpopulations while preserving protective ones (12–14). For a comprehensive overview, the molecular signatures, functional properties, and clinical relevance of the major neutrophil subpopulations identified in sepsis have been systematically summarized in **Table 1**.

**Table 1:** Neutrophil subsets in sepsis.

Subtypes	Species	Trial	Model	Functional properties	Clinical relevance	Ref
CD64^+^PD-L1^+^	Human	Clinical	Septic patients	Impaired activation and phagocytosis functions	Early identification of sepsis	(13)
CD64^+^CD123^+^	Human	Clinical	Septic patients	Impaired activation and phagocytosis functions	Early identification of sepsis	(13)
Lta4h^+^Sort^+^	Mouse	*In vivo*	CLP model	High expression of inflammatory cytokines	Sepsis liver dysfunction	(65)
CD177^+^	Mouse	*In vivo*	CLP model	Secrete large amounts of NETs	Sepsis lung injury	(66)
CXCR4^+^CD40^+^CD86^+^MHCII^+^	Human	Ex vivo	Neutrophils treated with eCIRP	Induce T cell differentiation into Th1 to release IFN-γ	Sepsis lung injury	(67)
S100A8/A9^hi^	Mouse	*In vivo*	CLP model	Secrete large amounts of S100A8/A9	Sepsis lung injury	(14)
CD66b^+^	Human	Clinical	Septic patients	Inhibit proliferation and activation of CD4^+^T cells	Sepsis immunosuppression	(12)
CXCR2^+^	Human	Clinical	Septic patients with immunosuppression	Migrate to lung and differentiate into the PD-L1^+^ subtype	Sepsis immunosuppression	(68)
PD-L1^+^	Mouse	Ex vivo	Neutrophils treated with LPS	Induce T cell differentiation into Tregs and accelerate T cell apoptosis	Sepsis immunosuppression	(69)
Siglec-F^+^	Mouse	*In vivo*	PICS model	Secrete large amounts of IL-10	Sepsis immunosuppression	(70)
CD200R^hi^	Mouse	*In vivo*	CLP model	Induce T cell differentiation into Tregs	Sepsis immunosuppression	(71)

## Crosstalk between neutrophil and other immune cells

The sophisticated intercellular interaction network is the core regulatory mechanism for maintaining organismal homeostasis, which achieves the dynamic balance of the microenvironment through complex signal transduction and material exchange (1). This cell communication system is not only the basic defense mechanism for organisms to respond to internal and external stimuli (such as pathogen recognition, injury repair, etc.), but its dysregulation has also been proven to be a key driving factor in the pathological cascade of sepsis (6).

### Neutrophil-mediated immune cell regulation

Recent studies have established that neutrophils significantly influence T cell function during sepsis, with neutrophil-mediated dysregulation of T cell immunity representing a key pathological mechanism underlying sepsis-associated immunosuppression (5). The specific mechanisms primarily include paracrine regulation and cell contact-dependent pathways. Neutrophils modulate T cell differentiation and promote immunosuppression through the release of cytokines or DAMPs. Research has identified a specific neutrophil subset in sepsis, characterized by high CD200R expression, that drives naïve CD4^+^ T cell differentiation into Tregs through the production of insulin-like growth factor 1 (IGF-1) (71). NETs have been found to induce metabolic reprogramming of naive CD4^+^ T cells, thereby promoting their conversion to Tregs and upregulating Foxp3 expression to enhance their immunosuppressive activity (72). Siglec-F^+^ neutrophils can secrete IL-10, which suppresses T cell activation and cytokine production (70). In the cell-contact-dependent regulatory pathway, programmed death ligand-1^+^ (PD-L1^+^) neutrophils can directly act on T cells through the programmed death (PD-1)/PD-L1 signaling axis, thereby inhibiting the function of CD4^+^ T cells and inducing their conversion to immunosuppressive phenotypes, ultimately promoting immunosuppression (68). High mobility group B1 (HMGB1) has been shown to activate TLR2 signaling, resulting in upregulation of PD-L1 expression in neutrophils. The subsequent PD-L1/PD-1 interaction triggers T cell apoptosis, contributing to lymphopenia and immunosuppression (73). Qi et al. demonstrated that lipopolysaccharide (LPS) upregulates neutrophil PD-L1 via the p38α/mitogen and stress-activated protein kinase 1 (MSK1) pathway. This PD-L1/PD-1 interaction mediates immunosuppression through three mechanisms: promoting naïve CD4^+^ T cell differentiation into Tregs, suppressing effector T cell function, and inducing T cell apoptosis (69). Cd66^+^ neutrophils have also been found to significantly inhibit T cell activation, but the underlying mechanism requires further investigation (12). Beyond immunosuppression, neutrophil-T cell crosstalk can also exacerbate inflammation. Jin et al. discovered that extracellular CIRP induces neutrophil differentiation into a novel functional subset (67). These cells exhibit a unique CXCR4^+^CD62L^-^CD40^+^CD86^+^MHCII^+^ phenotype and secrete high levels of IL-12, thus being named “antigen-presenting aged neutrophils” (APANs) (67). During sepsis, APANs bind to CD4+ T cells through their surface molecules CD40, CD86, and MHCII, promoting the differentiation of T cells into the Th1 subset and enhancing the release of interferon-gamma (IFN-γ). Subsequently, IFN-γ can promote NETosis in neutrophils, thereby exacerbating inflammatory response and organ damage (67). Notably, the interaction between neutrophils and macrophages plays a key role in the pathogenesis of sepsis-related lung injury. It was reported that neutrophil-derived exosomal miR-30d-5p exacerbates lung injury through a dual mechanism: activating the NF-κB inflammatory pathway in macrophages to promote IL-1β and tumor necrosis factor alpha (TNF-α) expression, and inducing macrophage pyroptosis, thereby synergistically amplifying pulmonary inflammatory cascades (74).

### Immune cell-mediated neutrophil regulation

Exosomal vesicles secreted by M2 macrophages have been demonstrated to downregulate the expression of chemokine receptors on neutrophils, thereby reducing neutrophil infiltration into the lungs and ameliorating sepsis-induced acute lung injury (75). During sepsis, CD300a^+^ mast cells and dendritic cells have been found to suppress the directional migration of neutrophils into the peritoneal cavity. Genetic or pharmacological disruption of CD300a in these immune cells significantly enhances neutrophil chemotaxis to the peritoneum, consequently improving the host’s ability to clear pathogens (76). Another study found that dendritic cells can accelerate neutrophil infiltration into the lungs by secreting large amounts of S100A8/A9, thereby exacerbating sepsis-induced lung injury (77). He et al. discovered that under septic conditions, natural killer (NK) cells significantly promote the secretion of chemokines such as CXCL1 and CXCL2 by specifically activating microglia, thereby facilitating abnormal infiltration of neutrophils into brain tissue and ultimately leading to irreversible neuronal damage (78). Recent studies have shown that the neutrophil-platelet interaction also plays a key regulatory role in the pathological process of sepsis. Stimulator of interferon genes (STING) activation can significantly upregulate the expression of P-selectin on the platelet surface, and then enhance the NETosis activation and the NETs release through the P-selectin/PSGL-1 ligand-receptor interaction, ultimately exacerbating sepsis-induced thrombosis (79). HMGB1 and miRNAs (miR-15b-5p/miR-378a-3p) carried by platelet exosomes can synergistically activate NETosis and promote NETs release, exacerbating sepsis-induced organ damage (80). Notably, studies have found that IFN-γ secreted by T lymphocytes can activate the JAK2/STAT1 signaling pathway in neutrophils, thereby upregulating their PD-L1 expression and promoting the generation of immunosuppressive neutrophils (81). These data suggest a potential feedback loop between neutrophils and T cells via the IFN-γ/PD-L1 axis: T cell-derived IFN-γ promotes PD-L1 expression on neutrophils, and upregulation of neutrophil PD-L1 in turn induces T cell dysfunction. This bidirectional interaction collectively aggravates the immunosuppressive state in sepsis. Based on this pathological mechanism, the development of dual inhibitors that simultaneously target neutrophil PD-L1 and T cell IFN-γ may provide a new therapeutic strategy for reversing sepsis-induced immune paralysis.

## Regulated cell death of neutrophil in sepsis

It is noteworthy that the aberrant activation of programmed cell death pathways in immune cells constitutes another critical pathological feature of sepsis (82, 83). Notably, neutrophil death not only leads to dynamic imbalances in neutrophil numbers but also triggers the abnormal release of their inflammatory mediators (e.g., TNF-α, IL-1β), resulting in the typical pathological process of “cytokine storm” (84). For example, studies have shown significant activation of anti-apoptotic pathways in neutrophils from both septic animal models and patients. This adaptation prolongs neutrophil survival, enhancing pathogen clearance and adaptive immunity initiation. However, under pathological conditions, persistently overactivated neutrophils accumulate in tissues and release abundant ROS and proteolytic enzymes. This pathological process drives microcirculatory dysfunction and tissue injury, representing a key mechanism of poor prognosis in sepsis (10, 85). Furthermore, hyperactivation of NETosis has been shown to play a pivotal role in the pathogenesis of sepsis-associated ARDS (86, 87). Clinical data show that compared with sepsis patients without concurrent ARDS, the levels of NETs in the serum of ARDS patients are significantly increased, with these concentrations positively correlating with Sequential Organ Failure Assessment (SOFA) scores (86, 87). NETosis-related genes have been identified as independent risk factors for predicting 28-day mortality (86, 88). Therefore, monitoring neutrophil death can provide preliminary yet valuable insights into the severity of impaired anti-infective immune responses, offering not only prognostic value but also novel interventional perspectives for sepsis management.

Over the past decade, the Nomenclature Committee on Cell Death (NCCD) has systematically refined the classification of cell death based on morphological, biochemical, and functional criteria (89). According to regulatory characteristics, cell death is now categorized into two primary types: accidental cell death (ACD) and regulated cell death (RCD). ACD refers to uncontrolled, acute cell death resulting from extreme physical, chemical, or mechanical insults. In contrast, RCD represents a highly conserved, molecularly orchestrated process that can be modulated by pharmacological or genetic interventions (89). Mechanistically, ACD constitutes a passive response to external damage, whereas RCD involves the cascade activation of precisely regulated signaling networks and effector programs. Of note, physiological RCD is specifically referred to as programmed cell death (PCD). Based on distinct molecular mechanisms, identified RCD subtypes include but are not limited to: apoptosis, necroptosis, pyroptosis, and NETosis (89). This article focuses on exploring the molecular characteristics and regulatory mechanisms of neutrophil death patterns during the pathological process of sepsis, aiming to provide a theoretical basis for the innovation of clinical treatment strategies.

## Neutrophil apoptosis in sepsis: delayed death and its drivers

Apoptosis is an actively regulated form of programmed cell death, precisely controlled by specific genetic programs (90, 91). Its fundamental purpose is the selective elimination of redundant, functionally impaired, or potentially harmful cells through highly ordered molecular mechanisms. Caspase (CASP) family proteins serve as the central regulators of apoptosis, with CASP3/7 acting as key executioners whose activation depends on the upstream caspase cascade. When cells receive lethal stimuli, apoptosis can be initiated via two primary pathways: the mitochondrial pathway and the death receptor pathway (89, 91). The mitochondrial pathway involves increased mitochondrial outer membrane permeability (MOMP), leading to cytochrome c release, formation of the apoptosome with Apoptotic protease activating factor 1 (Apaf-1), and subsequent activation of CASP9 and CASP3/7. The death receptor (e.g., Fas cell surface death receptor [FAS]/Tumor Necrosis Factor Receptor 1 [TNFR1]) pathway is triggered when death receptors bind their ligands, recruiting Fas-associated protein with death domain (FADD) to form the death-inducing signaling complex (DISC) and activating CASP8, which then cleaves CASP3/7. Both pathways ultimately converge on the activation of CASP3/7 to execute apoptosis (89, 91).

**Figure 3:**
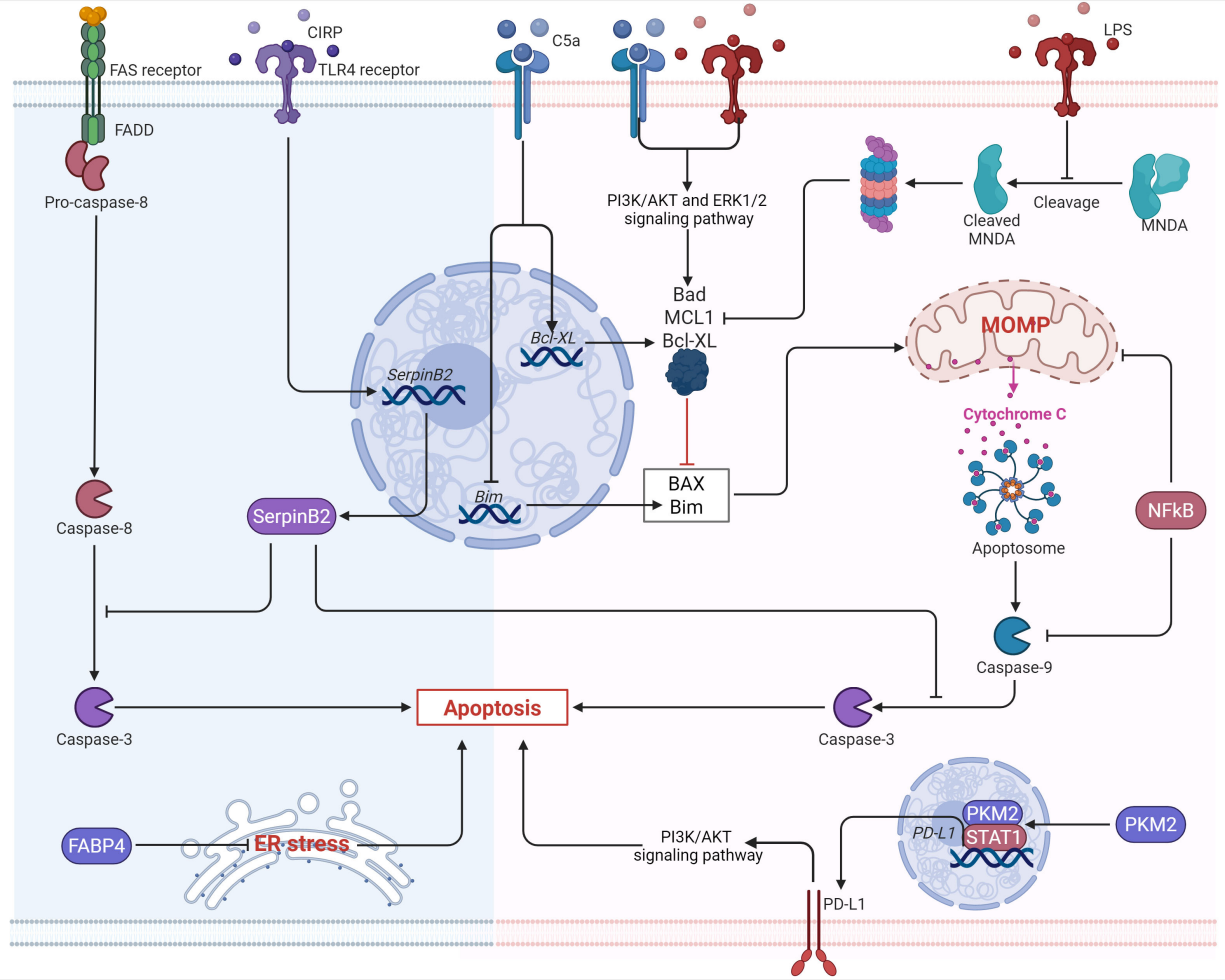
Apoptosis of neutrophil in sepsis. C5a upregulates Bcl-XL expression and inhibits Bim expression to suppress apoptosis. LPS inhibits neutrophil MDNA cleavage, blocking Mcl-1 ubiquitin−proteasome degradation and delaying apoptosis. LPS and C5a co-stimulate neutrophils to activate ERK1/2 and PI3K/Akt, thereby promoting Bad phosphorylation and inhibiting apoptosis. NF-κB activation inhibits apoptosis by stabilizing mitochondrial membrane potential and suppressing CASP9 activity. CIRP upregulates SerpinB2 expression through TLR4 receptor to inhibit CASP3 activation and apoptosis. FABP4 suppresses apoptosis by alleviating ERS. PD-L1 inhibits apoptosis by activating the PI3K/AKT signaling pathway. PKM2 translocates to the nucleus and forms a STAT1 complex, upregulating PD-L1 and inhibiting neutrophil apoptosis.

During sepsis, neutrophil apoptosis exhibits significant temporal dysregulation. This delayed apoptosis has been confirmed to correlate closely with disease progression and clinical outcomes (92, 93). Current evidence indicates that the septic microenvironment induces characteristic apoptosis inhibition in neutrophils through several key molecular mechanisms (**Figure 3**). First, the septic stimuli modulate the expression and activity of apoptosis-related proteins in neutrophils. C5a bidirectionally regulates the expression of neutrophil apoptosis-related proteins: it significantly upregulates the anti-apoptotic protein B-cell lymphoma-extra large (Bcl-XL) while inhibiting the expression of the pro-apoptotic protein Bcl-2-like protein 11 (BIM) (94). LPS stimulation can inhibit neutrophil myeloid nuclear differentiation antigen (MNDA) cleavage, thereby blocking the ubiquitin-proteasome degradation pathway of MCL-1 protein to delay neutrophil apoptosis (93). Additionally, combined stimulation with LPS and C5a activates both the extracellular signal-regulated kinase (ERK1/2) pathway and the phosphoinositide 3-kinase (PI3K)/AKT serine/threonine kinase (Akt) signaling axis in neutrophils, thereby promoting the phosphorylation and functional activation of the anti-apoptotic protein Bcl-2-associated agonist of cell death (Bad) (95). Second, the caspase (CASP) protein family serves as a crucial target for regulating neutrophil apoptosis. NF-κB activation inhibits apoptosis by stabilizing mitochondrial membrane potential and suppressing CASP9 activity (96). CIRP upregulates the expression of serine protease inhibitor B2 (SerpinB2) through a TLR4 receptor-dependent pathway, and this protein can inhibit the activation of CASP3, thereby effectively blocking the apoptotic pathway (97). Furthermore, the endoplasmic reticulum stress pathway plays a significant role in neutrophil apoptosis. Fatty acid binding protein 4 (FABP4) has been found to suppress apoptosis by mitigating excessive endoplasmic reticulum stress (ERS) in neutrophils (98). Finally, PD-L1 acts as a key regulator of neutrophil apoptosis. PD-L1 significantly inhibits apoptosis by activating the PI3K/AKT signaling pathway (92). Pyruvate kinase M2 (PKM2) translocates to the nucleus and forms a transcriptional complex with STAT1, markedly upregulating PD-L1 expression and thereby inhibiting neutrophil apoptosis (99). However, the precise molecular mechanism by which PD-L1 regulates neutrophil apoptosis still needs to be clarified through more in-depth experiments.

## Neutrophil necroptosis in sepsis: kinase-driven death and inflammatory role

Necroptosis represents a distinct form of programmed cell death that operates independently of the caspase protease system, instead relying on a precisely regulated kinase cascade for its execution (91, 100). Its activation primarily depends on receptors from the TNFR and TLR families, IFN signaling pathways, and intracellular RNA/DNA sensors. Upon activation, these receptors recruit receptor-interacting protein kinases (RIPK1 and RIPK3) to form the necrosome. RIPK3 subsequently phosphorylates the downstream effector protein mixed lineage kinase domain-like protein (MLKL), leading to MLKL oligomerization and translocation to the plasma membrane, ultimately resulting in membrane disruption and necroptotic cell death (91, 100).

Studies have demonstrated that necroptosis, as a key regulatory pathway of inflammatory cell death, contributes significantly to sepsis pathogenesis. Its inhibition markedly attenuates sepsis-induced damage in multiple organs, including the lung, kidney, and liver (101–103). Genetic knockout or pharmacological inhibition targeting the kinase activities of RIPK1 and RIPK3 not only effectively mitigates systemic inflammatory responses but also significantly improves organ function. This protective effect is particularly prominent in neonatal mouse models of sepsis, offering potential novel targets for the precise clinical treatment of sepsis (104). Wang et al. found that the LPS-TLR4 signaling axis suppresses RIPK1-RIPK3-MLKL necrosome formation through activation of TANK-binding kinase 1 (TBK1)/NF-κB kinase epsilon (IKKϵ) (105). Depletion or inhibition of TBK1 enhances the RIPK1-RIPK3-MLKL interaction, promoting neutrophil necroptosis and exacerbating inflammation. This indicates that the LPS-TLR4-TBK1 axis serves as a negative regulatory pathway for necroptosis, suggesting that targeting TBK1/IKKϵ could represent a novel strategy to mitigate infection-associated neutrophil death and inflammation (105).

## Neutrophil pyroptosis in sepsis: gasdermin-dependent pathway and inflammatory amplification

Pyroptosis is a programmed inflammatory cell death process mediated by gasdermin family proteins, primarily gasdermin D (GSDMD) and GSDME. Its molecular mechanism involves inflammatory caspases (CASP1/11 and human CASP4/5) cleaving GSDMD to generate the pore-forming N-terminal fragment (GSDMD-N), while GSDME is specifically activated by the apoptosis-related CASP3 (91, 106). The initiation of the CASP1-GSDMD pathway depends on the assembly of inflammasome platforms such as NLRP3 and Absent in Melanoma 2 (AIM2), whereas the CASP3-GSDME pathway is triggered by classical apoptotic signaling activation. Ultimately, plasma membrane pores formed by GSDMD-N/GSDME-N lead to cell death and the release of pro-inflammatory cytokines such as IL-1β and IL-18 (91, 106).

Studies demonstrated that the NLRP3 inhibitor MCC950 significantly alleviates oxidative stress in neutrophils, thereby suppressing pyroptosis and consequently mitigating lethal inflammatory responses while improving survival in septic mice (107). Ma et al. confirmed that LPS stimulation activates the CASP3-GSDME pyroptosis pathway in neutrophils, thereby mediating sepsis-induced lung injury. Interestingly, interference with GSDME expression in neutrophils shifted their death mode from pyroptosis to apoptosis without affecting neutrophil viability. However, the increased neutrophil apoptosis promoted macrophage efferocytosis, thereby alleviating systemic inflammation (108). N-acetyltransferase 10 (NAT10) acts as a key negative regulator of neutrophil pyroptosis in sepsis by suppressing the GSDMD-mediated pyroptosis pathway via the UNC-51-like kinases 1 (ULK1)-STING-NLRP3 axis. Mechanistically, NAT10 upregulates ULK1 protein expression by mediating N4-acetylcytidine (ac4C) modification of ULK1 mRNA, and ULK1 effectively inhibits the activation of the STING-interferon regulatory factor 3 (IRF3)-NLRP3 signaling pathway, consequently blocking GSDMD-mediated pyroptosis (109). In addition, clinical studies further revealed that the key regulatory genes of pyroptosis are significantly upregulated in peripheral blood neutrophils of sepsis patients, and their expression levels are closely related to the clinical prognosis of patients (110).

## NETosis in sepsis: regulatory pathways and induction of secondary cell death

NETosis is a regulated form of neutrophil cell death characterized by the release of NETs in response to infection or injury (91, 111). Activation of NETosis not only prevents the spread of pathogens such as bacteria by trapping or killing them but also promotes the release of DAMPs, thereby participating in the occurrence and progression of various diseases, including sepsis, cardiovascular diseases, and cancer (91, 111). The process involves several key steps. First, NADPH oxidase (NOX) promotes the generation of reactive oxygen species (ROS). Subsequently, ROS induce the degradation of cytoplasmic granules containing MPO and NE. Next, NE translocates into the nucleus and initiates histone cleavage, leading to chromatin decondensation. Concurrently, peptidylarginine deiminase 4 (PAD4) is activated, which promotes histone H3 citrullination and further facilitates chromatin decondensation. Finally, chromatin combines with granular proteins to form NETs, which are released into the extracellular space upon cell membrane rupture (91, 112).

**Figure 4:**
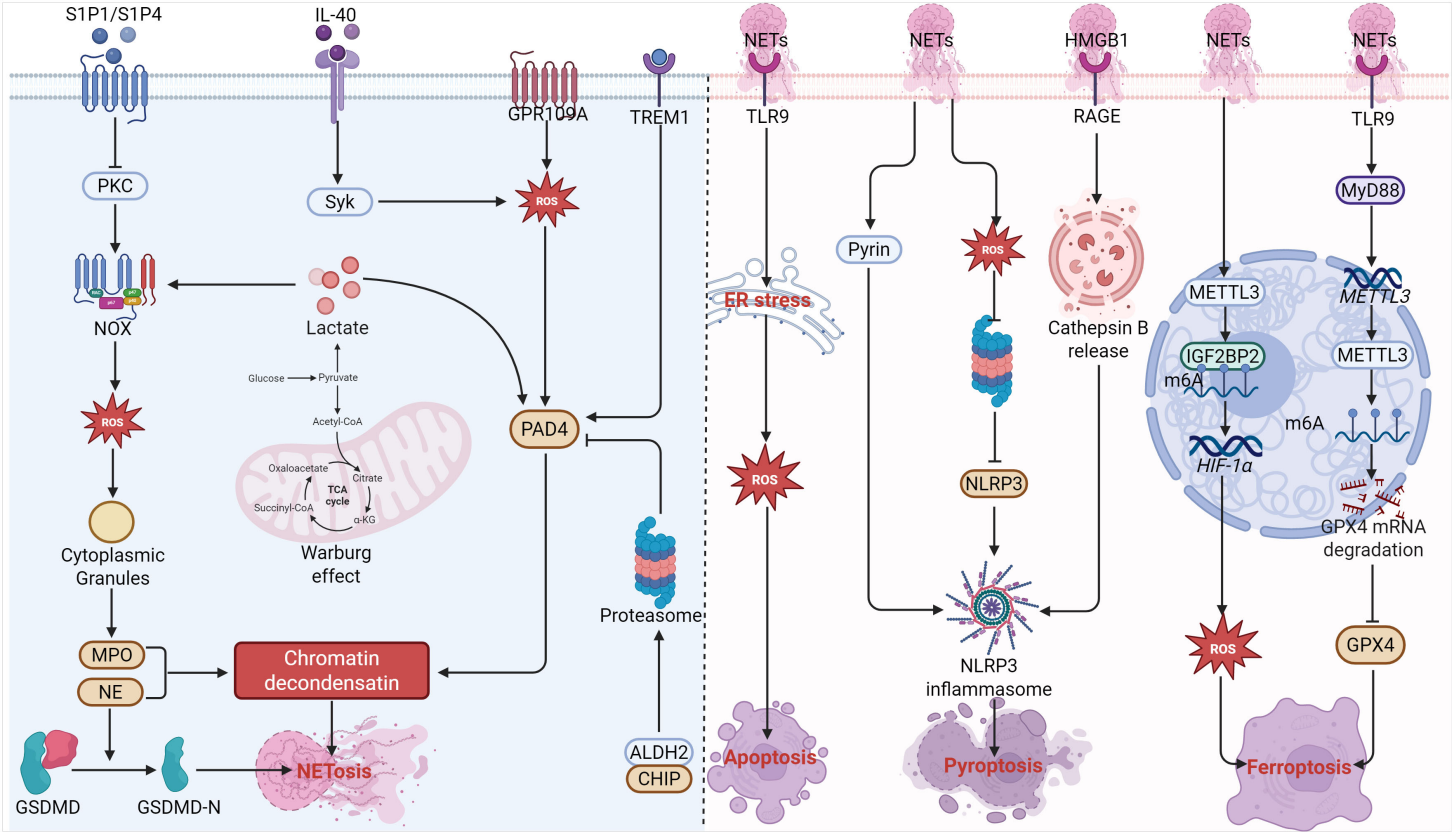
NETosis and NETs-related cell death in sepsis. ApoM binds to neutrophil surface receptors via its S1P1/S1P4, inhibiting PKC activation and NETosis. The Warburg effect increases lactate production to promote NETosis through NOX and PAD4 pathways. GPR109A reduces NETosis by inhibiting ROS-PAD4 signaling axis. IL-40 promotes NETosis by activating Syk-ROS-PAD4 signaling axis. ALDH2 binds CHIP to promote PAD4 degradation via the proteasome pathway and inhibit NETosis. TREM1 activates PAD4-dependent NETosis. NE cleaves GSDMD, which then creates membrane pores to promote NETosis and NET release. NETs activate TLR9-ERS-ROS axis to promote intestinal epithelial cell apoptosis. NETs promote macrophage pyroptosis through multiple mechanisms: activating Pyrin to accelerate NLRP3 inflammasome formation, promoting ROS production to inhibit proteasome-dependent degradation of NLRP3, and delivering HMGB1 that activates RAGE to promote cathepsin B release. NETs activate METTL3 to upregulate HIF-1α via the m6A-IGF2BP2 axis, promoting ROS accumulation and ferroptosis. NETs upregulate METTL3 via the TLR9/MyD88/NF-κB pathway. Subsequently, METTL3-mediated m6A modification then degrades GPX4 mRNA, thereby inducing ferroptosis.

In sepsis, apolipoprotein M (ApoM) has been found to bind to neutrophil surface receptors via its carried S1P1/S1P4, inhibiting the activation of protein kinase C (PKC), thereby effectively suppressing NETosis (113). Awasthi et al. found that neutrophils from septic patients exhibit significant activation of the Warburg effect, leading to increased lactate production. This excess lactate exacerbates cell death by promoting both NOX-dependent and NOX-independent NETosis pathways (114). G-protein-coupled receptor 109a (GPR109A) has been shown to significantly reduce sepsis-induced NETosis by inhibiting the ROS-PAD4 signaling axis (52). A study revealed that serum levels of IL-40 are significantly elevated in patients with septic shock, and its expression level is positively correlated with patient mortality. Further investigation demonstrated that IL-40 significantly promotes NETosis and stimulates the release of inflammatory factors and DAMPs by activating the neutrophil spleen tyrosine kinase (Syk)-ROS-PAD4 signaling axis, ultimately leading to MODS (115). Aldehyde dehydrogenase 2 (ALDH2) can enhance the ubiquitination of PAD4 by binding to the E3 ubiquitin ligase C-terminus of Hsc70-interacting protein (CHIP), thereby promoting rapid degradation of PAD4 via the proteasome pathway and effectively inhibiting NETosis (86). Loss of ALDH2 function leads to significantly increased NETosis and exacerbates sepsis-induced vascular endothelial injury (86). Abnormal activation of triggering receptor expressed on myeloid cells (TREM-1) on neutrophils has been identified as a key driver of excessive NETosis in sepsis, though its downstream molecular mechanisms remain incompletely elucidated (116). Additionally, the pyroptosis execution protein GSDMD not only mediates pyroptosis but also synergistically promotes NETosis by enhancing NETs release, forming a “pyroptosis-NETosis” positive feedback loop that further amplifies inflammatory responses. Specifically, during NETs formation, NE cleaves GSDMD, and the cleaved form of GSDMD then creates pores in the plasma membrane to facilitate NETs release (117). This study indicates that there is close crosstalk between multiple forms of neutrophil death, and synergistic targeted regulation of multiple cell death pathways is expected to become a new strategy for sepsis treatment (**Figure 4**).

Notably, NETosis not only accelerates neutrophil death but also activates pattern recognition receptors on other cells through the released NETs, triggering inflammatory signaling pathways (91, 111). This process induces additional cell death, further aggravates tissue injury, and forms an amplified inflammatory cascade, serving as a critical mechanism driving sepsis progression (**Figure 4**). Sun et al. discovered that excessively formed NETs during sepsis activate the TLR9-ERS-ROS axis, promoting intestinal epithelial cell apoptosis and inflammation, ultimately leading to disruption of the intestinal barrier (118). NETs release and subsequent excessive activation of macrophage pyroptosis are also important contributors to sepsis exacerbation. Mechanistically, NETs drive inflammasome activation and pyroptosis mainly through three signaling pathways. First, NETs directly activate Pyrin, promoting its recruitment of the associated speck-like protein containing a CARD (ASC) and CASP1 to accelerate inflammasome assembly. Second, NETs promote ROS generation in macrophages, inhibiting NLRP3 ubiquitination and thereby driving inflammasome formation. Third, HMGB1 contained in NETs can initiate inflammasome activation through the receptor for advanced glycation end products (RAGE)-cathepsin B signaling axis. These three pathways collectively constitute a NETs-dependent inflammation amplification network, exacerbating the pathological process of sepsis (119–121). Additionally, NETs can accelerate sepsis progression by activating ferroptosis. Studies have shown that NETs induce ferroptosis in alveolar epithelial cells by activating METTL3-mediated m6A modification. Mechanistically, METTL3 enhances HIF-1α expression through the m6A-IGF2BP2 axis, and this transcription factor then causes a metabolic shift toward increased glycolysis and decreased oxidative phosphorylation. This metabolic disorder leads to massive accumulation of intracellular ROS, ultimately inducing cellular ferroptosis (122). Another study revealed that NETs can activate the TLR9/myeloid differentiation primary response 88 (MyD88)/NF-κB signaling pathway to upregulate methyltransferase-like 3 (METTL3) expression; subsequently, METTL3-mediated m6A modification drives ferroptosis in alveolar epithelial cells by promoting the degradation of glutathione peroxidase 4 (GPX4) mRNA and downregulating this key antioxidant enzyme (123).

These studies collectively reveal that NETosis exacerbates sepsis injury by releasing NETs that activate multiple cell death programs, forming a cascade amplification effect. Effectively blocking this malignant signaling network remains a critical direction for future research.

## Targeting neutrophil during sepsis

Immune dysregulation driven by neutrophil dysfunction has emerged as a critical determinant of sepsis prognosis. A growing body of evidence indicates that aberrant neutrophil activity not only compromises pathogen clearance but also exacerbates tissue damage, thereby contributing to disease severity and poor clinical outcomes (5). In recent years, various immunomodulatory therapies have been shown to modulate neutrophil function and survival, with several related clinical trials currently underway. These findings have opened up a new avenue for sepsis treatment, shifting the focus from broad immunoregulation to more refined regulation of neutrophil biology (6). To date, intervention strategies targeting neutrophils primarily concentrate on two major directions: first, modulating their functional activation status (e.g., chemotaxis, phagocytosis, and respiratory burst), and second, regulating their cell death pathways (e.g., apoptosis, necroptosis, pyroptosis, and NETosis). Together, these approaches hold promise for restoring immune homeostasis and improving survival in septic patients.

## Modifying neutrophil function

Current evidence indicates that therapeutic strategies targeting neutrophil dysfunction in sepsis primarily focus on four key aspects: First, inhibiting the excessive release of NETs. Acetylsalicylic acid can effectively alleviate sepsis-induced coagulation dysfunction and improve microcirculatory perfusion by suppressing NETs release from neutrophils (124). Another study demonstrated that hesperetin modulates the ROS-autophagy signaling axis, thereby reducing NETs formation to protect intestinal barrier function and alleviate sepsis-associated intestinal injury (51). Yang et al. confirmed that curdione exerts lung-protective effects in sepsis models by interfering with platelet-mediated NETs formation (125). Second, reducing neutrophil-derived ROS production. Carbon monoxide (CO) therapy has been shown to inhibit ROS generation in neutrophils, thereby alleviating pulmonary oxidative stress and inflammatory responses, and ultimately protecting against sepsis-induced lung injury (126). The anti-inflammatory activity of pyquitinib in sepsis is mediated through its suppression of neutrophil-derived ROS generation (127). Third, enhancing neutrophil phagocytic function. For instance, CD5L and CD300ld have been found to promote pathogen clearance by enhancing neutrophil phagocytosis, thereby effectively controlling infections and delaying sepsis progression (128, 129). The ability of mesenchymal stromal cells to improve sepsis prognosis is attributed to their secretion of CXCL12, which enhances neutrophil-mediated bacterial clearance by boosting phagocytic activity (130). Fourth, regulating neutrophil migration. Exosomal vesicles secreted by M2 macrophages have been verified to reduce neutrophil infiltration into the lungs by downregulating the expression of the CXCR2 on neutrophils, thereby ameliorating sepsis-induced acute lung injury (75). Nintedanib mediates its protective effect against sepsis-induced lung injury by activating GRK2 to downregulate CXCR2, thus inhibiting neutrophil recruitment to the lungs (131). Additionally, failure to clear infections due to impaired neutrophil migration represents another key mechanism driving sepsis progression. By upregulating CXCR2 expression, paroxetine can maintain neutrophil migration to infection foci, thereby promoting efficient pathogen clearance which in turn mitigates sepsis-induced lethal inflammation (132). Another study demonstrated that trimetazidine activates the AMP-activated protein kinase (AMPK)/Nuclear factor erythroid 2-related factor 2 (Nrf2) pathway to upregulate CXCR2, a mechanism that accelerates pathogen clearance by enhancing neutrophil migration to the heart, ultimately alleviating myocardial dysfunction in sepsis (133). In addition, several clinical trials are exploring the feasibility of improving sepsis prognosis by modulating neutrophil function. Researchers have attempted to use GM-CSF to enhance neutrophil phagocytic capacity in order to improve infection control in ICU patients with sepsis (ClinicalTrials.gov ID NCT01653665). The NE inhibitor Sivelestat sodium has also been investigated for its potential to improve the clinical outcomes of patients with sepsis-associated ARDS (ClinicalTrials.gov ID NCT05672472). Overall, targeting neutrophil dysfunction holds significant potential for improving sepsis treatment outcomes.

Notably, translational feasibility, specificity, and adverse effects remain key challenges for neutrophil−targeted therapies in sepsis. Most preclinical studies use young, healthy animals with early intervention, whereas patients are often older, present late, and have comorbidities; only a few agents (e.g., GM−CSF, sivelestat) have entered clinical trials with inconsistent results. Achieving neutrophil selectivity is difficult because surface receptors (e.g., CXCR2) are shared with other immune cells, and broad inhibition of neutrophil activation may affect other immune cells. Given the dual protective and pathogenic roles of neutrophils, complete blockade of functions such as NETosis or apoptosis risks secondary infections or prolonged inflammation. Thus, future strategies should aim to “reprogram” rather than “abolish” neutrophil activity through timed, context−dependent, or locally targeted approaches.

## Regulating neutrophil cell death

Currently, sepsis treatment strategies targeting neutrophil death are primarily achieved through the following two pathways. First, drugs directly regulate neutrophil death. The small-molecule pyroptosis inhibitor MCC950 can block the pyroptotic process of neutrophils in sepsis, thereby significantly reducing systemic inflammatory responses and improving the survival rate of septic mice (107). Adla-1, an agonist of ALDH2, has been shown to inhibit the abnormal activation of NETosis caused by *ALDH2* mutations, which in turn reduces pulmonary inflammatory responses and delays the development of sepsis-induced ARDS (86). Another study found that shikonin promotes the neutrophil apoptosis during sepsis by upregulating the expression of CASP3 and MCL-1, thereby alleviating inflammatory reactions and improving sepsis-associated lung injury (134). Second, drugs inhibit NETs-induced death of other cells by blocking NETs release. For example, reduced NETs release mediated by alpha-linolenic acid has been identified as a key mechanism for inhibiting macrophage death in sepsis, and this pathway constitutes the core link underlying its ability to alleviate lung inflammation and delay sepsis progression. Mesenchymal stem cells have also been shown to inhibit NETs release, thereby reducing NETs-induced endothelial cell ferroptosis and exerting tissue-protective effects in sepsis-induced lung injury (135).

Although numerous drugs have demonstrated favorable protective effects in sepsis models by regulating neutrophil functional activation or death, most studies remain in the animal experimental phase, with their clinical efficacy and translational potential yet to be clarified. Additionally, the existing intervention approaches (such as exosomes, Chinese herbal extracts, and stem cells) generally suffer from insufficient cell and tissue targeting. Therefore, future research should focus on the specific molecular alterations of neutrophils in sepsis and strive to develop more targeted therapeutic strategies.

## Conclusion and future directions

Neutrophils, the most abundant circulating immune cells and first responders to infection, undergo profound functional and cell death alterations during sepsis. Targeting these cells to modulate host immunity has therefore become a key direction in sepsis research. Multiple interventional strategies have been applied in sepsis models, effectively mitigating aberrant immune responses by regulating neutrophil activity or death. Although these approaches remain preclinical, they offer significant prospects for immune management in sepsis. However, a major limitation is that most mechanistic insights come from animal studies, predominantly murine models. Significant species differences exist between human and mouse neutrophils, including variations in receptor expression, NETosis regulation, and cellular lifespan. Moreover, many drugs that showed efficacy in murine sepsis have failed in human trials. Thus, preclinical findings must be interpreted with caution. Greater emphasis should be placed on human neutrophil research using humanized mouse models and ex vivo patient samples.

In summary, further elucidating the specific mechanisms of neutrophils in sepsis is essential. By systematically deciphering the molecular regulatory networks of neutrophils under septic conditions and developing novel targeted therapeutic strategies based on these insights, it will be possible to optimize interventions for immune dysregulation in sepsis and provide new directions for clinical treatment.
